# 5-Lipoxygenase facilitates healing after myocardial infarction

**DOI:** 10.1007/s00395-013-0367-8

**Published:** 2013-06-28

**Authors:** Nadja Blömer, Christina Pachel, Ulrich Hofmann, Peter Nordbeck, Wolfgang Bauer, Denise Mathes, Anna Frey, Barbara Bayer, Benjamin Vogel, Georg Ertl, Johann Bauersachs, Stefan Frantz

**Affiliations:** 1 Department of Internal Medicine I, University Hospital Würzburg, Würzburg, Germany; 2 Comprehensive Heart Failure Center, University of Würzburg, Oberdürrbacher Straße 6, 97080 Würzburg, Germany; 3 Department of Cardiology and Angiology, Medizinische Hochschule Hannover, Hannover, Germany

**Keywords:** Myocardial infarction, Extracellular matrix remodeling, Inflammation, Lipoxygenase

## Abstract

Early healing after myocardial infarction (MI) is characterized by a strong inflammatory reaction. Most leukotrienes are pro-inflammatory and are therefore potential mediators of healing and remodeling after myocardial ischemia. The enzyme 5-lipoxygenase (5-LOX) has a key role in the transformation of arachidonic acid in leukotrienes. Thus, we tested the effect of 5-LOX on healing after MI. After chronic coronary artery ligation, early mortality was significantly increased in 5-LOX^−/−^ when compared to matching wildtype (WT) mice due to left ventricular rupture. This effect could be reproduced in mice treated with the 5-LOX inhibitor Zileuton. A perfusion mismatch due to the vasoactive potential of leukotrienes is not responsible for left ventricular rupture since local blood flow assessed by magnetic resonance perfusion measurements was not different. However, after MI, there was an accentuation of the inflammatory reaction with an increase of pro-inflammatory macrophages. Yet, mortality was not changed in chimeric mice (WT vs. 5-LOX^−/−^ bone marrow in 5-LOX^−/−^ animals), indicating that an altered function of 5-LOX^−/−^ inflammatory cells is not responsible for the phenotype. Collagen production and accumulation of fibroblasts were significantly reduced in 5-LOX^−/−^ mice in vivo after MI. This might be due to an impaired migration of 5-LOX^−/−^ fibroblasts, as shown in vitro to serum. In conclusion, a lack or inhibition of 5-LOX increases mortality after MI because of healing defects. This is not mediated by a change in local blood flow, but through an altered inflammation and/or fibroblast function.

## Introduction

After an acute myocardial infarction (MI), a complete restoration of the infarcted tissue would be ideal. Indeed, some species can restore organs or limbs in total; however, mammalians are restricted to incomplete reparation [8]. Cardiac adaption after MI occurs in different phases [8, 9]: an inflammatory phase in the first hours, the early healing phase in the first days, and finally the remodeling phase. Impaired healing after MI can directly lead to fatal problems such as left ventricular rupture; subsequently, maladaptive scar formation may also be responsible for adverse left ventricular remodeling. Unfortunately, knowledge of healing after MI and potential pharmacologic targets to improve healing are scarce [9]. Inflammation plays a pivotal role in all the above-mentioned phases. Leukotrienes are regulators of inflammation and thus potential targets to influence healing after MI [23]. Furthermore, leukotrienes are activated after cardiac injury. Leukotriene B4 is increased after experimental myocardial infarction in the rat myocardium [26] and in patients with cardiac ischemia [4].

Leukotrienes comprise a family of products of the 5-lipoxygenase (5-LOX) pathway of arachidonic acid metabolism binding to outer plasma membrane receptors [23]. Leukotrienes are necessary for the function and migration of virtually all subgroups of leukocytes. Moreover, they influence fibroblasts, increase contractility and proliferation of smooth muscle cells and are also important for vascular permeability. So far leukotrienes have been associated with asthma, cancer, and atherosclerotic vascular lesions (for a more detailed review, see [23]). Populations with gene variants that increase leukotriene production have higher incidences of stroke and myocardial infarction [17]. Since all of these mechanisms mentioned above are important for cardiac healing, we investigated the function of leukotrienes after MI using mice that lack 5-LOX and therefore leukotriene production as we have previously shown [1]. We had the hypothesis that leukotrienes would be necessary for proper function of inflammatory cells, perfusion, and extracellular matrix remodeling and therefore healing would be blunted in 5-LOX^−/−^ mice.

## Methods

### Animals and surgery

Male 5-LOX^−/−^ and wildtype (WT) mice of 8–12 weeks old with a bodyweight of 20–28 g were purchased from Jackson Laboratory (Bar Habor, Maine, USA) [1]. Mice underwent left coronary artery ligation to induce MI, as described recently [18]. A subgroup of WT mice received placebo or Zileuton (50 mg/kg bodyweight, po bid [3]). The governmental Standing Committee on Animal Research approved the animal study protocol. Infarct size was measured 3 days after MI using Evans blue/TTC staining as recently described [12].

### Echocardiographic analysis

Ultrasound analyses were performed by a single researcher experienced in rodent echocardiography (Toshiba, Aplio) blinded to mouse genotype at days 1 and 3 immediately before sacrificing the animals, as recently described [10]. All animals after MI were serially imaged. Using two-dimensional short-axis imaging, endocardial borders were traced at end-systole and end-diastole with a prototype off-line analysis system (NICE, Toshiba Medical Systems, the Netherlands). Measurements were performed at the mid-papillary muscle level. The end-systolic (smallest) and end-diastolic (largest) cavity areas were determined. Using the end-systolic and -diastolic areas, fractional area changes were calculated [(end-diastolic area − end-systolic area)/end-diastolic area]. From two-dimensionally targeted M-mode tracings, end-diastolic diameter and end-systolic diameter were measured. Fractional shortening was calculated. Only animals with a large infarct size and a heart rate >450/min were included.

### Bone marrow transplantation (BMT)

Bone marrow transplantation was performed as previously described [12].

### Perfusion measurements

Myocardial in vivo perfusion was noninvasively quantified using an arterial spin-labeling magnetic resonance imaging (MRI) technique. MRI was performed on day 3 after coronary artery ligation using a Bruker Biospec 70/20 scanner (Bruker Biospin, Germany). The scanner has a horizontal bore with a field strength of 7.05 T, equipped with a G60 microscopy gradient system, a customized radio frequency transmit coil in birdcage design, and dedicated surface coil for optimized signal detection. MRI was done under inhalation anesthesia using room air with 1.5 % isoflurane and 0.5 % oxygen. The imaging protocol started with an ECG-triggered multi-slice short-axis cine-FLASH (fast low-angle shot) with an image resolution of 0.234 × 0.234 and slice thickness of 1 mm, contiguously covering the whole heart. Myocardial infarct size was determined as the area of myocardium with significant thinning, akinesia, or dyskinesia for every slice.

Spin-labeling perfusion MRI was performed as described before [28], running a segmented ECG-gated inversion recovery snapshot FLASH to quantify T1 relaxation time. Image acquisition was performed in the late diastole, therefore covering the period of maximum myocardial perfusion while at the same time avoiding heart motion. T1 measurements were done in a midventricular short-axis view (field of view 30 × 30 mm, slice thickness 2 mm). Sixteen signal averages were performed to increase signal-to-noise ratio. Data post-processing was done using Matlab, deriving perfusion maps with a nominal resolution of 0.234 × 0.234 × 2 mm. Perfusion values were then calculated for the intramural remote and infarcted myocardium separately voxel by voxel, excluding areas prone to artifacts/partial volume effects such as the subendocardial layer.

### Biochemical and molecular measurements

Myocardial RNA isolation and real-time PCR measurements were performed as previously described [11] with commercially available TaqMan probes for 18S, TNF (tumor necrosis factor), and collagen 1 (Applied Biosystems, Foster City, CA, USA). RNA samples were normalized to 18S rRNA as described [22]. Matrix metalloproteinase 9 (MMP-9) was measured in duplicate by a commercial ELISA (R&D Systems, Abingdon, UK) according to the manufacture’s protocol as described with tissue from the remote region after myocardial infarction [22].

### FACS analysis

Cell suspensions from individual hearts were prepared by digestion with collagenase type 2 and protease type XIV (Sigma Aldrich, Germany) as recently described in detail [2]. For flow cytometry, stainings were performed with up to 10^6^ trypan blue-negative cells in 50 μl of PBS/0.1 % BSA/0.02 % NaN_3_. Fcγ II/III receptors were pre-blocked by incubation with saturating amounts of cell culture supernatant of the clone 2.4G2 (anti-Fcγ receptor II/III monoclonal antibody). Fluorochrome-conjugated or biotinylated mAbs were added after blocking (15 min, 4 °C). Cells were stained with CD45 FITC, CD11b PE, Ly6G Alexa 647, and Ly6C Cy5. After washing, the cells were studied on a FACS Calibur (BD Biosciences, Heidelberg) cytometer for four-color analysis. Data were collected from the live gate using forward/side scatter plots. For myocardial cell suspensions, the leukocyte gate was initially defined by staining the whole cell suspension for CD45 expression. Data were processed and analyzed by CellQuest (BD Bioscience, Heidelberg) or FlowJo (Tristar, Ashland OR) software.

### Immunohistochemistry

Formalin-fixed sections of mouse myocardium and in vitro fibroblasts were prepared in the standard manner. Primary antibodies used were alpha sarcomeric actinin (αSA, ab9465, Abcam, UK), fibroblast specific protein 1 (FSP-1, PAK0073, Linaris, Germany), alpha smooth muscle actin (αSMA, 6198, Sigma, Germany), or a non-immune immunoglobulin. Cells were labeled by the sequential application of the primary antibody and a peroxidase–anti-peroxidase complex followed by labeling (Vectastain ABC Kit, Vector Laboratories, Burlingame, CA, USA). The slides were washed, dehydrated and mounted for light microscopy. Vimentin was stained accordingly with a Cy3-labeled antibody (Sigma, Germany) for fluorescent microscopy.

### Fibroblast isolation

Primary cultures of 5-LOX^−/−^ and WT hearts were successfully established from unoperated left ventricles (*n* = 4 in each group). Left ventricular tissue was minced into small pieces, digested as described and transferred into culture flasks [27]. Culture media consisted of Dulbecco’s modified Eagle’s media (DMEM, Lonza, Cologne, Germany) supplemented with 10 % fetal bovine serum (FBS).

### Fibroblast functional assays

Commercially available functional fibroblast assays were used according to the manufactures’ protocols. All tests were performed after overnight starvation. Specifically, collagen production in the supernatants was measured with the sircol assay (Sircol, biocolor, BioCat GmbH, Heidelberg, Germany) and a Mouse PINP EIA (AC-33F1, Immunodiagnostic Systems, Frankfurt, Germany), adhesion using the CytoSelect 48-Well Cell Adhesion Assay (Cell Bioloabs, BioCat GmbH, Heidelberg, Germany) with wells coated with fibronectin, collagen I, collagen IV, laminin I, fibrinogen, and BSA as negative control. For the migration assay, serum starved cells were seeded into the upper compartment of a fibronectin-coated transwell tissue culture insert (Thincert cell culture insert for 24 well plates, 8 μm pore size, Greiner Bio-One, Kremsmünster, Austria). 10 % FBS served as chemoattractant and was placed in the coated lower chamber of each well. Following 5 h of incubation, cells were stained with calcein and counted. Collagen gel deformation was measured with a cell contraction assay (Cell Biolabs, BioCat GmbH, Heidelberg, Germany). 1 × 10^6^ cells in 650 μl collagen gel lattice were cultured for 2 days. As negative control, cells were pretreated with 10 mM 2,3-butanedione monoxime (BDM) for 1 h before initiation of contraction. The change of gel size in millimeters was measured after 24 h of contraction with ImageJ software (ImageJ 1.44p, National Institutes of Health, USA).

### Statistical analysis

All replicate data are expressed as mean and standard error of mean. Mortality rates were compared using a log rank test. Absolute differences among groups were compared using a two-way ANOVA adjusted by the Fisher rule. Statistical significance was achieved when two-tailed *p* < 0.05. Statistical analyses were carried out using StatView statistics program (Abacus Concepts, Inc., Berkley, CA, USA).

## Results

### Mortality post-MI in mice with impaired leukotriene production

**Fig. 1:**
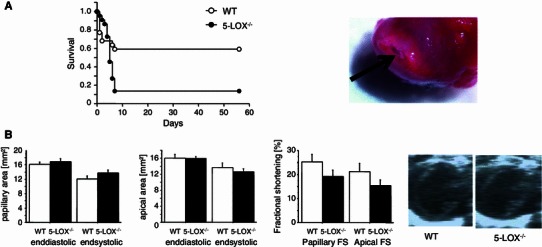
Mortality and left ventricular architecture after MI. **a** Mortality was significantly increased in LOX^−/−^ mice after MI. Most mice died due to left ventricular rupture (see *arrow* in the representative picture). **b** This was not due to early changes in left ventricular remodeling (echocardiography, day 3 after MI and representative 2D-echo images of LOX^−/−^ and WT animals)

Mortality was significantly increased in 5-LOX^−/−^ when compared to WT mice after chronic coronary artery ligation (survival, *n* = 44, WT vs. 5-LOX^−/−^, 59 vs. 14 %, *p* < 0.01, see Fig. 1). Most of the 5-LOX^−/−^ mice died due to left ventricular rupture within the first week, indicating a healing defect in 5-LOX^−/−^ mice. Infarct size measured by Evans blue/TTC staining was not different between the groups (WT vs. 5-LOX^−/−^, infarct size, *n* = 10, 25.8 ± 4.8 vs. 24.9 ± 1.6, area at risk 32.0 ± 3.1 vs. 32.4 ± 4.0, *p* = ns). To confirm these effects with an independent loss of function approach, we also treated WT mice with zileuton, a leukotriene antagonist. Increased mortality up to day 3 after MI due to left ventricular rupture could be reproduced in zileuton-treated animals after chronic MI (*n* = 15, survival 27 %, *p* < 0.01 vs. WT).

In summary, inhibition or deficiency of 5-LOX causes high mortality due to healing defects after MI.

### Left ventricular remodeling in 5-LOX^−/−^

To see if changes in the left ventricular architecture are responsible for the healing defects, animals underwent echocardiography at day 3 after permanent coronary artery ligation, a time point before left ventricular rupture takes place. All measurements were recorded at the mid-papillary level, which shows changes in the dimensions of the surviving non-infarcted myocardium, as well as on the apical level depicting changes in scar formation. There were no differences in left ventricular dimensions in both groups (see Table 1; Fig. 1). Thus, there were no obvious differences in macroscopic left ventricular architecture early after MI responsible for left ventricular rupture.

**Table 1:** Echocardiographic measurements at day 3 after MI

	Sham		MI	
	WT	KO	WT	KO
*N*	7	6	9	8
Heart rate (1/min)	604 ± 14	623 ± 24	572 ± 14	590 ± 12
Papillary				
End-diastolic diameter (mm)	4.0 ± 0.1	3.8 ± 0.2	5.1 ± 0.1**	5.0 ± 0.1**
End-systolic diameter (mm)	2.4 ± 0.1	2.3 ± 0.2	4.5 ± 0.2**	4.2 ± 0.2**
Fractional shortening (%)	41 ± 2	40 ± 3	12 ± 2**	16 ± 1**
End-diastolic wall thickness, posterior wall (mm)	0.7 ± 0.03	0.7 ± 0.03	0.7 ± 0.05	0.7 ± 0.04
Apical				
End-diastolic diameter (mm)	3.9 ± 0.1	3.7 ± 0.2	4.9 ± 0.1**	5.0 ± 0.1**
End-systolic diameter (mm)	2.3 ± 0.2	2.1 ± 0.2	4.5 ± 0.2**	4.4 ± 0.1**
Fractional shortening (%)	42 ± 2	43 ± 2	11 ± 2**	11 ± 1**

Data are mean ± SEM, *n* indicates number of animals studied

** *p* < 0.001 MI vs. Sham

### Microvascular perfusion after MI

**Fig. 2:**
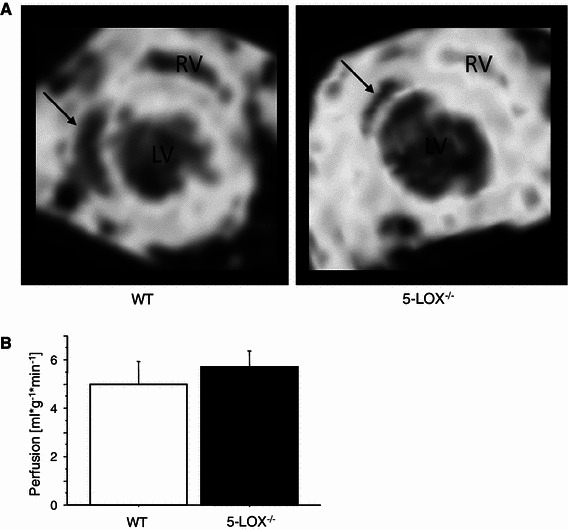
Left ventricular perfusion after MI. **a** Myocardial perfusion maps acquired by arterial spin-labeling MRI 3 days after myocardial infarction. Representative midventricular short-axis slices are shown for WT and LOX^−/−^ mice with signal intensity encoding spatial perfusion (*LV* left ventricle, *RV* right ventricle, *arrows* indicate the non-perfused infarction area). **b** Myocardial perfusion was not different between the genotypes

Leukotrienes increase contractility in smooth muscle cells and can thus influence vascular tone and perfusion of the non-infarcted and scar area after MI. One potential explanation for left ventricular rupture could be a perfusion mismatch caused by 5-LOX inhibition. Thus, spin-labeling perfusion MRI was performed for the quantitative measurement of myocardial perfusion in vivo. Regional perfusion was individually calculated for all voxels in plane and then averaged for the respective regions of interest covering infarcted and remote myocardium. Representative MRI maps illustrating spatial myocardial perfusion are shown in Fig. 2. Mean perfusion did not differ in the remote myocardium between both groups (WT vs. 5-LOX^−/−^, *n* = 9, 5.00 ± 1.9 vs. 5.72 ± 1.3 ml/g/min, *p* = ns). In the scar region, perfusion was diminished to a similar extent in both genotypes (WT vs. 5-LOX^−/−^, 0.53 ± 0.3 vs. 0.84 ± 0.5 ml/g/min, KO vs. WT, *p* = ns). Infarct size was not different between the groups. Therefore, disturbed myocardial perfusion cannot be held responsible for the healing defect in 5-LOX^−/−^ mice.

### Role of LOX in inflammation after MI

Leukotrienes influence attraction and function of almost all leukocyte subgroups. On the other hand, leukocytes are also an important source of leukotriene production. Since the inflammatory response is important for healing, we next had the hypothesis that a reduction in inflammatory cell activation would be responsible for healing defects after MI in 5-LOX^−/−^ mice.

**Fig. 3:**
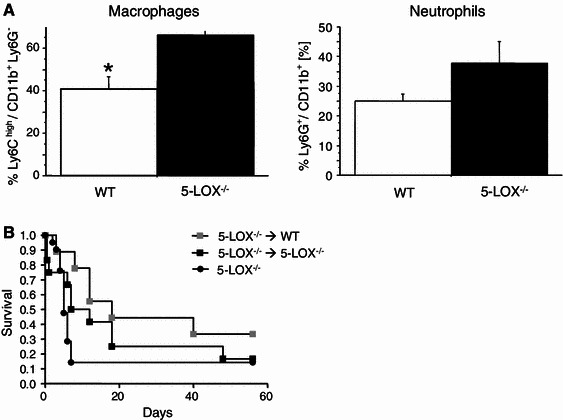
Inflammation after MI. **a** As determined by FACS analysis of the infarcted myocardium 3 days after MI, there was an increase of pro-inflammatory macrophages, but not of neutrophils after MI in LOX^−/−^ mice. **b** To investigate if healing defects are due to functional impairment of LOX^−/−^ bone marrow-derived inflammatory cells, we generated chimeric mice by transplanting WT bone marrow cells in lethally irradiated KO animals. Left ventricular rupture could still be observed in 5-LOX^−/−^ mice reconstituted with WT bone marrow (*p* = ns)

TNF was decreased in 5-LOX^−/−^ mice after MI (WT vs. 5-LOX^−/−^, *n* = 16, 1.1 ± 0.3 vs. 0.7 ± 0.4 AU, *p* < 0.05). CD11b^+^ Ly6G^+^ granulocytes and CD11b^+^ Ly6G^−^ monocytes/macrophages were studied by FACS analysis in the infarcted myocardium at day 3 after MI. Analysis of the monocyte differentiation marker Ly6C on the CD11b^+^ Ly6G^−^ subset revealed a significant increase of the pro-inflammatory monocyte subset in 5-LOX^−/−^ mice (see Fig. 3, WT vs. 5-LOX^−/−^, *n* = 17, 41 ± 6 vs. 66 ± 2 %, *p* < 0.01), whereas the relative proportion of neutrophils was not altered (see Fig. 3, WT vs. 5-LOX^−/−^, 25 ± 2 vs. 38 ± 7 %, *p* = ns).

Since monocyte subset composition was changed and monocytes/macrophages are a major source of leukotriene production, we tested in the next step the functional consequence of 5-LOX deficiency in bone marrow-derived cells in vivo. We generated chimeric mice by transplanting WT bone marrow cells in lethally irradiated 5-LOX^−/−^ animals. 5-LOX^−/−^ animals transplanted with 5-LOX^−/−^ bone marrow served as controls. Previously we had documented successful reconstitution in chimeric mice [12]. Six weeks later, these mice were subjected to myocardial infarction. However, the high rates of left ventricular rupture could still be observed in 5-LOX^−/−^ mice reconstituted with WT bone marrow (see Fig. 3). This indicates that a functional change of bone marrow-derived 5-LOX^−/−^ cells is not responsible for healing defects after MI in 5-LOX^−/−^ mice. Nevertheless, increased infiltration of pro-inflammatory cells remains a plausible mechanism for left ventricular rupture.

### Role of LOX in extracellular matrix remodeling after MI

**Fig. 4:**
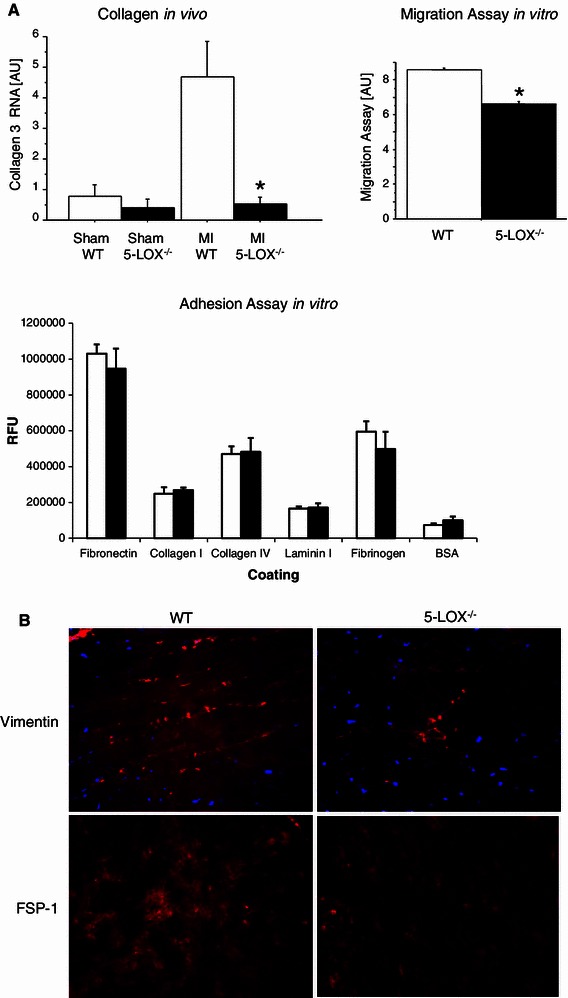
Extracellular matrix remodeling. **a** Collagen RNA was significantly decreased in the scar region 3 days after MI in vivo. In vitro migration, but not adhesion of 5-LOX^−/−^ fibroblasts was significantly impaired when compared to WT fibroblasts. **b** In vivo, fibroblasts of the border zone after MI were reduced as detected by vimentin and FSP1 immunohistochemistry (red staining, ×40 magnification)

Besides its influence on perfusion and inflammation, leukotrienes are involved in collagen metabolism, another important factor for healing after MI. To test whether collagen metabolism is altered after MI in vivo, we measured MMP and collagen expression in the scar region after MI. MMP-9 was not different between the genotypes (WT vs. 5-LOX^−/−^, *n* = 12, 0.6 ± 0.3 vs. 0.2 ± 0.1 ng/100 μg protein, *p* = ns). However, collagen RNA was significantly reduced in 5-LOX^−/−^ mice (see Fig. 4). In a second step, we tried to elucidate the mechanisms of altered extracellular matrix remodeling in vitro. For this purpose, we isolated WT and 5-LOX^−/−^ fibroblasts. In vitro collagen production as measured in tissue culture supernatants was not different between the genotypes (acid- and pepsin-soluble collagens: WT vs. 5-LOX^−/−^, *n* = 8, 35 ± 5 vs. 29 ± 7 AU, *p* = ns; procollagen type I amino terminal peptide: WT vs. 5-LOX^−/−^, 1.31 ± 0.03 vs. 1.21 ± 0.03 ng/ml, *p* = ns). Fibroblast contractility was the same in both groups (WT vs. 5-LOX^−/−^, 23.7 ± 3.5 vs. 24.7 ± 6.2 %, *p* = ns). Markers of fibroblast to myofibroblast phenoconversion (αSMA, FN) were also not altered in vitro. An assay testing the adhesion to fibronectin, collagen I, collagen IV, laminin, fibrinogen, and albumin also revealed no differences between the genotypes (see Fig. 4). However, migration of 5-LOX^−/−^ fibroblasts to serum was significantly impaired (see Fig. 4). Accordingly, the number of fibroblasts in vivo was significantly reduced as measured by FSP-1 and vimentin staining (WT vs. 5-LOX^−/−^, 103.3 ± 19.8 vs. 42.9 ± 8.6 AU, *p* < 0.05). Taken together, there was a significant reduction in collagen RNA and infiltration of fibroblasts in 5-LOX^−/−^ after MI in vivo, most likely due to a reduction in the migration capacity of 5-LOX^−/−^ fibroblasts.

## Discussion

The arachidonic acid cascade is involved in different mechanisms important for healing and remodeling after MI. Leukotrienes influence the inflammatory reaction, fibroblast activation and local perfusion. To investigate the role of leukotrienes after chronic MI, we used 5-LOX^−/−^ mice that are unable to produce leukotrienes. After chronic MI, roughly 80 % of the mice died due to left ventricular rupture, a sign for a healing defect.

There are several potential mechanisms for this finding that were thoroughly investigated by us.

### Changes in microcirculation

Several clinical studies have shown that the preservation of flow in the infarct area reduces left ventricular dilatation and subsequent remodeling and leads to an improvement in regional and global systolic function [24, 25]. Leukotrienes are important for blood vessel contraction. Thus, one potential mechanism for impaired healing in 5-LOX^−/−^ mice could be a mismatched blood flow to the diseased myocardium, e.g., through an inadequately low blood supply to the scar. To test if this is the case, we spatially measured myocardial perfusion by arterial spin-labeling MRI. The technique allows one to noninvasively quantify local perfusion; it has recently been matched with perfusion values determined by invasive microsphere examination, proving accurateness of this measurement methodology [28]. However, we could demonstrate that neither in the scar nor in the remote region perfusion is significantly altered between WT and 5-LOX^−/−^ mice.

### Changes in inflammation

The inflammatory reaction is of paramount importance for healing after MI. Within 24 h, inflammatory signals recruit neutrophils to the infarct zone, shortly thereafter monocytes. Inflammatory cells have then multiple functions in this early healing phase such as degradation of extracellular matrix constituents and scavenging of dead cardiac myocytes and their debris. After approximately 5 days, monocytes/macrophages and endothelial cells coordinate angiogenesis and promote blood supply to form a solid scar. About 2–3 weeks after MI, monocytes/macrophages disappear and the granulation tissue matures into a solid scar [9, 19, 29, 30].

Considering the importance of inflammation for healing and the influence of leukotrienes on inflammatory cells, healing in 5-LOX^−/−^ mice might potentially be impaired due to changes in infiltration pattern and function of inflammatory cells. Indeed, when we checked the infiltration of inflammatory cells in the digested scar region by FACS analysis 3 days after chronic MI, i.e. shortly before left ventricles rupture, we found an increase of the pro-inflammatory monocyte subset in the infarcted myocardium. Although counterintuitive, this finding is in line with previous results in 5-LOX^−/−^ mice [1] and may be mediated by an alternative cyclooxygenase-dependent pathway in the absence of LOX [13].

As outlined above, monocytes/macrophages are central for the coordination of extracellular matrix degradation and reparation, which could potentially explain the observed healing defects. To see if a functional alteration of 5-LOX^−/−^ in inflammatory cells might be responsible for these effects, we lethally irradiated 5-LOX^−/−^ mice and reconstituted them with either WT or 5-LOX^−/−^ bone marrow. However, in both chimeric mouse strains, mortality was high due to left ventricular rupture and not different between groups.

This indicates that leukocyte-derived 5-LOX is not crucial for the healing defects. However, considering the increase in pro-inflammatory monocytes/macrophages, the altered inflammatory response is still a plausible mechanism for the phenotype.

### Extracellular matrix remodeling

Obviously, adequate healing depends on extracellular matrix remodeling [21]. This may be exemplified by the fact that a system of proteolytic enzymes, the MMPs, and their inhibitors, the tissue inhibitors (TIMPs) of MMPs are increased after MI [5]. Targeted deletion of MMP 2, 9, or TIMP-3 decreases the incidence of rupture and attenuates left ventricular enlargement after MI in mice [6, 15, 16]. However, the role of LOX in healing is controversial. While healing is improved in a few experimental models after LOX inhibition, e.g. after bone fracture [20], we found impaired healing after MI. There are a few in vitro reports linking 5-LOX^−/−^ to fibroblast function. 5-LOX products induce the synthesis of extracellular matrix proteins as well as collagenase, indicating that they participate directly in extracellular matrix metabolism during inflammation. 5-LOX is known to be an active chemoattractant for fibroblasts and to regulate fibroblast activity in in vitro wound healing assays [14]. To test whether 5-LOX^−/−^ fibroblasts are functionally impaired, we established cultures of WT and 5-LOX^−/−^ cardiac fibroblasts. Indeed, 5-LOX^−/−^ cardiac fibroblasts had impaired migration in vitro. Accordingly, collagen production and fibroblast density in the myocardial granulation tissue were diminished in vivo. Thus, the severe healing defect in 5-LOX^−/−^ mice after MI may be mediated by an attenuated fibroblast function.

### Clinical significance

Gene variants that increase 5-LOX activity are associated with an increased risk of MI due to its influence on atherosclerosis [17]. Carotid intima-media thickness was increased in carriers of two variant alleles of the 5-lipoxygenase promoter [7]. However, genetic data for adverse healing and remodeling after MI are lacking. Leukotriene inhibitors are extensively used in patients with asthma as a clinical standard treatment after corticosteroids. Yet, adverse cardiovascular effects have not been reported. This might be due to the fact that asthma in combination with leukotriene inhibitor treatment is not common in patients with acute myocardial infarction and chronic heart failure. Yet, a careful analysis of the existing data is warranted.

## Conclusion

A lack or inhibition of 5-LOX increases mortality after myocardial infarction due to cardiac healing defects. This is not mediated by changes of local blood flow, but by an increase of pro-inflammatory cells and an altered fibroblast function with consecutive changes in extracellular matrix remodeling. The clinical significance of these findings has to be readdressed in clinical studies using leukotriene inhibitors.
